# Difficult vs. Severe Asthma: Definition and Limits of Asthma Control in the Pediatric Population

**DOI:** 10.3389/fped.2018.00170

**Published:** 2018-06-19

**Authors:** Amelia Licari, Ilaria Brambilla, Alessia Marseglia, Maria De Filippo, Valeria Paganelli, Gian L. Marseglia

**Affiliations:** Department of Pediatric, Fondazione IRCCS Policlinico San Matteo, University of Pavia, Pavia, Italy

**Keywords:** asthma control and severity, severe asthma, difficult to treat asthma, asthma, treatment adherence, severe asthma risk factors

## Abstract

Evaluating the degree of disease control is pivotal when assessing a patient with asthma. Asthma control is defined as the degree to which manifestations of the disease are reduced or removed by therapy. Two domains of asthma control are identified in the guidelines: symptom control and future risk of poor asthma outcomes, including asthma attacks, accelerated decline in lung function, or treatment-related side effects. Over the past decade, the definition and the tools of asthma control have been substantially implemented so that the majority of children with asthma have their disease well controlled with standard therapies. However, a small subset of asthmatic children still requires maximal therapy to achieve or maintain symptom control and experience considerable morbidity. Childhood uncontrolled asthma is a heterogeneous group and represents a clinical and therapeutic challenge requiring a multidisciplinary systematic assessment. The identification of the factors that may contribute to the gain or loss of control in asthma is essential in differentiating children with difficult-to-treat asthma from those with severe asthma that is resistant to traditional therapies. The aim of this review is to focus on current concept of asthma control, describing monitoring tools currently used to assess asthma control in clinical practice and research, and evaluating comorbidities and modifiable and non-modifiable factors associated with uncontrolled asthma in children, with particular reference to severe asthma.

## Introduction

Asthma is one of the most common chronic diseases affecting all age groups, with up to 20% of children aged 6–7 years experiencing severe wheezing episodes within a year (1). Childhood asthma is currently defined as a clinical umbrella syndrome encompassing symptoms such as wheezing, shortness of breath, chest tightness, and cough, ranging in severity from mild symptoms to life-threatening attacks (2). In this context, several pathophysiologic components (traits) have been identified in children such as airflow limitation, eosinophilic airway inflammation, airway infection and impaired airway defenses, some of which being treatable (2). The primary goal of asthma management and treatment is to achieve the control of symptoms and underlying airway inflammation, aiming at minimizing the risk of future asthma attacks and medication-related side effects, and preventing the progression of obstructive lung damage during growth and then later in life (3).

International guidelines emphasize the importance of evaluating asthma control, rather than asthma severity, in order to guide asthma management decisions (3–5). Asthma control is defined as the degree to which manifestations of the disease are reduced or removed by therapy (6, 7). Two components of asthma control are identified in the guidelines: symptom control and risk. Symptom control domain refers to daytime symptoms and nighttime symptoms (i.e., cough, wheeze, exercise limitation), reliever use (short-acting β2 agonists, SABA) for the treatment of symptoms, and deviation from normal levels of activity (i.e., playing, sleeping, attending work or school), preserving normal or near-normal lung function, and meeting patient and parent expectations. Future risk of poor asthma outcomes domain includes preventing severe asthma attacks (requiring systemic corticosteroids, emergency medical treatment or hospitalization), loss of lung function or impairment of normal lung growth in childhood, and adverse effects caused by medication use (3–7). Asthma severity reflects the intrinsic “activity” of the disease and should be typically assessed both prior to beginning treatment with controller medications, and then at regular intervals to determine degree of responsiveness to treatment. Thus, asthma severity is usually defined from the level of treatment needed to achieve and maintain adequate control (7).

Over the past decade, the definition and the tools of asthma control have been substantially implemented so that the majority of children with asthma have their disease well controlled with standard therapies. However, a small subset of asthmatic children (<5% of all pediatric asthma) still requires maximal therapy to achieve or maintain symptom control and experience considerable morbidity (8–11). The identification of the factors that may contribute to the gain or loss of control in asthma is essential in differentiating children with difficult-to-treat asthma from those with severe asthma that is resistant to traditional therapies. The aim of this review is to focus on current concept of asthma control, describing monitoring tools currently used to assess asthma control in clinical practice and research, and evaluating comorbidities and modifiable and non modifiable factors associated with uncontrolled asthma in children, with particular reference to severe asthma.

## Assessment of asthma control

Evaluating asthma control starts with taking a detailed history of frequency of asthma symptoms, sleep disturbances, limitation of activity, and frequency of reliever use in the past 4 weeks. Treatment issues (adherence to therapy and inhaler technique) as well as comorbidities (i.e., nasal disease, eczema, food allergy, obesity, etc.) and factors that may complicate care should be also periodically reviewed (3).

Asthma attacks are characterized by an acute worsening of asthma symptoms in response to a trigger such as allergen exposure, viral infection, environmental irritants (including pollution and cigarette smoke) or a combination of these. Given that asthma attacks can occur also on the background of seemingly good control and normal lung function (12), the concepts of acute asthma attacks and asthma baseline control, although overlapping, are not the same.

### Symptom control

Common tools for monitoring asthma available in a clinical practice setting can be distinguished in subjective measures, such as asthma diaries and questionnaires-based composites asthma scores, and objective measures, including spirometry, bronchial hyperreactivity tests and biomarkers of airways inflammation (7).

#### Subjective measures

Subjective measures of asthma control include asthma diaries and asthma symptom questionnaires; moreover, visual analog scale (VAS) may be introduced in assessing patient's airways obstruction perception. These tools were initially introduced in clinical research; nevertheless, they are spreading also in clinical practice, since they enable the physicians to strictly monitor their patients' symptoms trends. Each of these tools presents pros and cons, that are briefly reported also in Table 1.

**Table 1:** Subjective measures of asthma control.

	**Pros**	**Cons**
*Asthma Diaries*	Do not depend on patient recall Monitor asthma-related events in real time	They can be retrospectively filled in Each diary collects large amount of data Lack of a standardized method to account for outcome data
*Asthma Symptom Questionnaires*		
ACQ, ACT, cACT, TRACK	Changes in composites asthma scores correlate to clinical deterioration and to the need of step-up therapy Provide data easy to analyze	Depend on patient recall Focus on a small-time window
CASI	Identify differences between patients who could seem similar only on the basis of their clinical symptoms Useful in clinical research to test impact of additional treatment in the context of standard care	Need for additional studies to validate the application of this tool Its role in clinical practice is unclear It does not include some relevant aspects of disease control
APGAR	Provides a wider spectrum of information than other questionnaires, about features that can be crucial in therapeutic planning It is linked to a care algorithm, which can help the physician to undertake a therapy	Need for additional studies to validate the application of this tool in clinical practice or in clinical research
*VAS*	Quick and feasible tool for “real-life” monitoring VAS <6 is a reliable marker of uncontrolled asthma Provides information about symptom perception	Need for additional studies to validate the application of this tool in clinical practice or in clinical research

*ACQ, Asthma Control Questionnaire; ACT, Asthma Control Test; APGAR, Activities, Persistent, triGGers, Asthma medications, Response to therapy; cACT, Childhood Asthma Control Test; CASI, Composite Asthma Severity Index; TRACK, Test for Respiratory and Asthma Control in Kids; VAS, Visual Analog Scale*.

Asthma diaries are created to obtain data regarding asthma-related events on a daily basis. So far asthma diaries validated for clinical practice or for clinical trials are the Pediatric Asthma Diary (PAD) (13), the Pediatric Asthma Caregiver Diary (PACD) (14), the daytime and nocturnal asthma symptom diary scales (15) and the Asthma Control Diary (ACD) (16). Asthma diaries collect data day-to-day, so that they do not depend on patient recall. This feature enables the physician to monitor asthma-related events in real time and to promptly adjust therapy. The main concern regarding paper diaries is represented by the risk of missing large amounts of data, since this kind of diaries can be retrospectively filled-in by the patient, either with invented data or with false data. In order to solve this problem, the diaries should be administered day-by-day through telematics-primed device, for instance telephone-administered diaries or online diaries (17–19). This solution has been already adopted in allergic rhinitis (20): as a matter of fact, the MASK-rhinitis study collected symptoms referred by patients, by means of a user-friendly smartphone application; so similar “apps” might be developed in order to be administered to asthmatic patients. Each asthma diary collects a large amount of data for each patient, so another concern about this tool regards the way to evaluate and analyse data derived from asthma diaries in clinical trials. Mixed model analyses should be adopted in order to report the large amount of longitudinal data collected by asthma diaries (21). Furthermore, there is not a standardized method to account for outcome data from asthma diaries: some studies used either “symptom-free days” (22) or “symptom days” (23) so as to obtain information about cost-benefit analysis. Other studies reported the proportion of patients with at least one event or the rate of annual events (24). Both these methods reveal the efficacy of medication in the population level, although a small number of patients may be responsible for a large number of events. Another method to report diaries-derived data is “time to first event” (25), which can estimate the impact of treatment on disease progression.

Asthma symptom questionnaires are intended to monitor asthma-related events over a period between 1 and 4 weeks. They can be administered only to patients (i.e. Asthma Control Questionnaire–ACQ; Asthma Control Test–ACT), or both to patients and their caregivers (i.e., Childhood Asthma Control Test–ACT), or only to caregivers (i.e., Asthma Therapy Assessment Questionnaire–ATAQ; Test for Respiratory and Asthma Control in Kids–TRACK). Their items usually include the frequency and impact of daytime symptoms, nocturnal symptoms, limitation of normal activities; some questionnaires include also questions about exercise induced-dyspnea (cACT and TRACK) and just one questionnaire evaluate also a measure of lung function (ACQ) (7). From all these data, each questionnaire elaborates a composite score, which express the level of asthma control. Four questionnaires (ACQ, ACT, cACT, TRACK) provide a cut-off value that enables the physician to distinguish between uncontrolled vs. controlled asthma, while ACT and ATAQ have a cut-off value to identify poorly controlled asthma (7). In clinical practice it may be useful to monitor for each patient how the answer to a specific question changes, so as to understand whether the disease is under control or not. Some changes in composites asthma scores correlate to clinical deterioration in asthma control and, consequently, to the need of a step-up of therapy (26). In clinical trials asthma questionnaires are more useful than asthma diaries because their provided data are more easily analyzable than those derived from asthma diaries. As a matter of fact, they have been used to assess the efficacy of different treatments (27, 28).

The answers given to asthma questionnaires depend on patient recall. Moreover, the questionnaires focus on a small-time window, which can not entirely reflect the level of disease control, because patients could improve their adherence to therapy just before clinical visit, recent events or recent period of bad control may bias reporting of the whole recall period and the trend of disease attacks can be inconstant through different seasons. In c-ACT, that is administered both to patients and to their caregivers, usually children give lower scores to asthma control than those given by their parents; in addition to that, some studies demonstrated that the correlation between c-ACT score and forced expiratory volume in the first second (FEV_1_) and forced vital capacity (FVC) is stronger in newly diagnosed asthma, while it becomes weaker in patients who undergo treatment for asthma (29).

VAS is a measurement instrument designed as 10 cm–long ruler, on which the patient is asked to put a mark corresponding to his symptom perception, considering that “0” represents “severe airflow obstruction perception,” while “10” represents “no respiratory symptoms.” VAS has been already introduced in some allergic conditions (such as rhinitis) as a quick, achievable instrument to monitor in “real-life” setting whether the disease is under control or not (30). Recently VAS has been suggested as a useful tool also for evaluating asthma symptoms perception: there are some evidence that a VAS <6 is a reliable marker of uncontrolled asthma in adults (31) and that, in children with bronchial obstruction, poor VAS scores well correlate with respiratory function decrease; moreover, after bronchodilation test, children with initial bronchial obstruction usually report a significant improvement in VAS scores (32). However, the perception of dyspnea has been little studied in severe pediatric asthma (33) and additional studies are needed to validate the application of this tool in clinical research and then in clinical practice.

Composite Asthma Severity Index (CASI) is a subjective measure based on multiple aspects of asthma severity, such as impairment, risk and treatment. It accounts for five domains of disease, which include symptoms (day symptoms and night symptoms), controller medication use, lung function tests and asthma attacks. The final score can go from 0 to 20: the higher it is, the more severe the disease is quantified (34). CASI estimates asthma severity by means of the level of medication needed to obtain the present clinical situation, in addition to impairment features and future risk measures. This peculiarity enables the examiner to identify differences between patients who, otherwise, could seem similar only on the basis of their clinical symptoms. Therefore, it is useful for clinical research, since it is sensitive in discriminating among treatment groups even in a well-controlled population, and it can detect the effect of a specific intervention in addition to guidelines-based treatment (35). However, this score needs to be validated on a larger population and its feasibility in clinical practice is unclear. Finally, it does not include some relevant aspects of disease control (i.e. quality of life and economic costs of asthma) (35).

The Asthma APGAR is a newly introduced tool for primary assessment of asthma. The acronym “APGAR” stands for “Activities, Persistent, triGGers, Asthma medications, Response to therapy,” it consists in a sequence of questions regarding not only the number of asthma attacks, the presence of diurnal or nocturnal symptoms or the limitation of normal activities due to asthma symptoms, but also the triggers of attacks, the way and the frequency with which the patients take their medicine and whether they think their medicines work or not. Its main distinctive feature is that it gathers a wider spectrum of information than other questionnaires: it mainly focuses on some peculiar aspects of the disease, which are not investigated by means of other questionnaires, but which can be pivotal for tailoring therapy (36). APGAR's questionnaire is also linked to a care algorithm, which is thought to help the physician to undertake a therapy, while all of the other questionnaires so far adopted only give the physician a simple score. Further evidences are needed to validate this tool as a reliable instrument in clinical research and/or in clinical practice.

Beyond electro-monitoring devices, mobile phone-based apps, gadgets and wearable devices, another tool assessing the degree of asthma disease control may be represented by Telemedicine. According to the American Telemedicine Association definition, it consists in “the remote delivery of health care services and clinical information using telecommunications technology” (37). It makes use of internet monitoring, text messages, email reminders. Its efficacy in controlling asthma is still unclear, and it may be cost-effective in rural communities or in underserved areas.

#### Objective measures

No objective gold-standard measure is currently available to diagnose asthma. Among objective measures commonly used to diagnose and monitor asthma, we can identify lung function tests, airway hyperresponsiveness (AHR) tests (Table 2) and biomarkers of airways inflammation.

**Table 2:** Obejctive measures of lung function.

	**Pros**	**Cons**
Spirometry	Objective, noninvasive, helpful for diagnosis and follow-up FEV_1_/FVC ratio can predict asthma-related morbidity and mortality even when FEV_1_ is still normal FEF_25−75_ is a sensitive indicator of small airway obstruction and a better indicator of a response to bronchodilators	It relies on patients' ability to carry out the test (unlikely applicable in children younger than 6 years old) Children may have briefer exhalation times and/or lower lung volumes than adults, consequently, their FEV_1_/FVC ratio may result normal even in case of airways obstruction Its great variability makes it unreliable as exclusive tool to assess airflow obstruction
PEF	Useful information about obstruction in the large central airways	The test is extremely effort-dependent
AHR	Objective, replicable, useful for diagnosis Exercise-induced bronchoconstriction is more specific than other provocation tests in detecting asthma among pediatric population	Unlikely applicable in young children It relies on patients' ability to carry out the test

*AHR, airway hyperresponsiveness; FEF_25-75_, forced expiratory flow rate over 25–75% of the FVC; FEV_1_, forced expiratory volume in the first second; FVC, forced vital capacity; PEF, peak of expiratory flow*.

Lung function tests, particularly spirometry, are objective, noninvasive, and extremely helpful in the diagnosis and follow-up of patients with asthma. Examination of the FVC, FEV_1_, and forced expiratory flow rate over 25%−75% of the FVC (FEF_25−75_) is a reliable way to detect baseline airway obstruction. An obstructive airflow pattern is defined by a reduced FEV_1_ (<0.80), a reduced FEV_1_/FVC ratio (normally >0.75 to 0.80, and usually >0.90 in children) and a concavity of the expiratory flow volume loop during a spirometry test (38). FEV_1_/FVC ratio is the most sensitive tool to assess the airflow obstruction and it is related to asthma severity; as a matter of fact, it can predict asthma-related morbidity and mortality even when FEV_1_ is still normal (39). However, younger children may have briefer exhalation times and/or lower lung volumes than adults, so that their FEV_1_ may be comparable to their FVC and, consequently, their FEV_1_/FVC ratio may result normal even in case of airways obstruction (40). FEF_25−75_ has been proposed as a sensitive indicator of small airway obstruction and a better indicator of a response to bronchodilators and AHR than either FEV_1_ or FVC; it can be considered normal when it is ≥70% of predicted FEF_25−75_ (41). Unfortunately, its great variability makes it unreliable as exclusive tool to assess airflow obstruction. In patients affected by asthma, it decreases earlier than other spirometry parameters and is able to predict long-term asthma persistence (42). Documentation of reversibility of air flow obstruction following inhalation of a bronchodilator is central to the definition of asthma. After bronchodilation, an improvement of at least 12% and 200 mL in the FEV_1_ is considered a positive response and is indicative of reversible air flow obstruction; however, in children, an improvement of 10% may be adequate to indicate significant improvement. It has been proved that the persistence of a significant degree of bronchodilator responsiveness despite regular treatment according to guidelines may represent a marker of worse asthma control (43, 44). Higher bronchodilator reversibility has been added as an additional independent risk factor for asthma attacks in both adults and children in 2018 update of the Global Strategy for Asthma Management and Prevention (3). Besides, reversible or partially reversible airflow obstruction seems to be a distinctive spirometry feature of pediatric severe asthma clinical phenotypes, as demonstrated by the results from severe asthma cohort studies (45, 46).

Although spirometry is still considered the gold standard for use in diagnosing and monitoring change in airway function in patients with asthma, other modalities may have particular application in both younger and older children. The Forced Oscillation Technique (FOT) is a lung function modality based on the application of an external oscillatory signal in order to determine the mechanical response of the respiratory system. The method is noninvasive and requires minimal patient cooperation, which makes it suitable for use in young pediatric patients. In the context of asthma, FOT may be more sensitive than spirometry in identifying disturbances of peripheral airways and assessing the level of asthma control or the effectiveness of therapy at the long term (47). Further research is required to determine the exact role of FOT as an objective monitoring tool in pediatric asthma.

Measuring peak of expiratory flow (PEF) may provide some useful information about obstruction in the large central airways. An exaggerated variability in PEF (morning-to-evening more than 20%) can confirm the diagnosis of asthma; however, the test is extremely effort-dependent and should not be used alone to diagnose asthma. Variability in PEF was suggested as an indicator of poor asthma control (48); however, studies evaluating the efficacy of PEF monitoring for improving various outcome measures in childhood asthma have yielded conflicting results (49–51).

AHR can be demonstrated either by direct inhaled agents (i.e., methacholine) or by indirect stimulants (i.e., exercise). Provocation tests with direct inhaled agents result positive if they cause a 20% fall in baseline FEV_1_, but they are not routinely used in pediatric population (52). Exercise challenge testing consists in assessing the FEV_1_ variations after 6–8 min of treadmill exercise; a decrease in FEV_1_ of more than 10% is diagnostic of exercise induced bronchoconstriction (53). There is some evidence that exercise-induced bronchoconstriction is more specific than other provocation tests in detecting asthma among pediatric population (54).

Airway inflammation is the hallmark of disease pathophysiology in asthma. Currently bronchoscopy, bronchial biopsy, and bronchoalveolar lavage (BAL) are considered the gold standard to assess airway inflammation and remodeling in asthma; however, the invasiveness of these diagnostic methods limits their use in pediatric age in daily clinical practice. Over the last 10 years, there has been an explosion of interest in the research of non-invasive biomarkers to assess of airway inflammation. Biomarkers for asthma have potential utility for distinguishing the inflammatory and/or molecular pattern or “endotype,” predict and monitor responsiveness to specific treatments, and assess of the risk of disease progression (55). Thus, the identification of non-invasive methods to study and monitor disease inflammation represents a relevant challenging field of research in childhood asthma. Most of the current established biomarkers available in clinical practice are related to allergic eosinophilic (Th2-high) inflammation. These include blood or sputum eosinophils, serum IgE, and fractional exhaled nitric oxide (FeNO) (55). Single and combination biomarkers are now being recommended for use in the assessment of children with asthma, in particular with severe asthma, and have been extensively reviewed elsewhere (55). Among these, fractional exhaled NO (FeNO) monitoring has a validated role in current clinical practice as a biomarker useful in asthma diagnosis, monitoring control, and predicting asthma attacks (56, 57). Its measurement is simple, safe, and well tolerated, and it has been standardized in school-aged children (58, 59). In pediatric asthma, FeNO is now recognized as a surrogate marker of eosinophilic airway inflammation and it is used to identify children with allergic asthma that are likely to respond to ICS treatment (60). Multiple studies have demonstrated that an increased FeNO value at baseline or increasing FeNO values during ICS reduction accurately predict an asthma attack (60). According to the cut-off values published in the American Thoracic Society (ATS) guidelines, a FeNO of > 35 ppb suggests a likely response to ICS, while a FeNO of <20 ppb in children indicates a less likely responsiveness to ICS treatment (61). Although FeNO-guided treatment has been associated with significantly fewer asthma attacks and lower attack rate than treatment based on current guidelines in childhood asthma, it not routinely recommended for the general asthma population at present (62). Identifying the populations most likely to benefit from FeNO-guided treatment, as well as establishing the optimal frequency of monitoring FeNO, still require further studies.

### Risk factors for poor asthma outcomes

The evaluation of symptom control should be always combined with the assessment of risk factors of adverse outcome in children with asthma, both at diagnosis and periodically thereafter.

First of all, having uncontrolled asthma symptoms, experiencing more than 1 episode of asthma attack in last year and/or admission to intensive care unit for asthma are the main independent risk factors for future attacks (63–67); other risk factors that are potentially modified have been recently updated and include: (i) high SABA use (more than 3 canisters/year) (68) and inadequate inhaled corticosteroids (ICS) therapy (not prescribed, poor adherence, incorrect inhaler technique) (69, 70); (ii) low FEV_1_ (even if normal spirometry does not exclude severe asthma in children) (71, 72) and higher bronchodilator reversibility (44); (iii) viral infections (rhinovirus and other respiratory viruses), allergen exposure in atopic children, tobacco smoke exposure and outdoor air pollution (65). Eosinophilic airway inflammation has been associated with risk of attacks that can be prevented with corticosteroid treatment (2). Blood eosinophils and FeNO have been identified as indirect measures of eosinophilic airway inflammation both in adults and children. These reliable biomarkers may provide a better perspective on risk of attacks and the likely response to treatment with corticosteroids than traditional physiological measures as lung function and asthma symptoms (2). However, blood eosinophilia and elevated FeNO have been recognized as risk factors for acute attacks mainly in adults with allergic asthma taking ICS (3). Overall, it has also been recently established that the presence of any of these mentioned conditions increases the risk of asthma attacks, even if the patient has few asthma symptoms (3).

The severity of asthma attack is a risk marker of both subsequent attacks and mortality from asthma (73). A recent revision of risk factors for severe asthma attacks identified nonwhite race, psycho-social stress and obesity as additional clinical predictors in children (67). Furthermore, cadherin-related family member 3 (CDHR3) has been identified as a novel susceptibility gene for recurrent severe asthma attacks in children ages 2–6 years: in particular, variants of CDH3 seem to alter the integrity of airway epithelium and subsequently promote entry and replication of respiratory viruses (74). However, with the exception of history of one recent severe attack, no current clinical or biological markers has been shown to be highly predictive of severe asthma attacks in children (67). Finally, the recent UK National Review of Asthma Deaths (NRAD) identified the conditions at high risk for death from asthma: (i) a single severe attack; (ii) recent discharge from hospital after an acute asthma attack; (iii) use of hospital urgent care facilities in the previous year; (iv) utilization of more than 6 canisters of SABA/year; and (v) failure to attend follow-up appointments^1^. Thus, an asthma attack (even one) should be carefully considered a significant immediate red flag signaling a high risk of future attacks and asthma deaths, in particular in primary care. Moreover, considering that NRAD reported that around 60% of those who died from asthma were classified as “mild to moderate,” the definition of severity by level of treatment should need to be questioned. All these mentioned factors should be added to the conventional definitions of risk as “high risk factors” with the aim to improve care and hopefully reduce the number of deaths.

Early growth characteristics such as pre-term birth, low birth weight and greater infant weight gain have been recently added to the list of risk factors of poor asthma outcomes, all being determinants of airflow limitation and associated with increased risk of childhood asthma (3, 75).

Children with uncontrolled asthma, in particular if treated with high-dose ICS and oral corticosteroids (OCS), may experience medication side-effects (i.e. local and systemic side-effects) (76, 77). The risk factors for medication side-effects include frequent use of oral steroids, long-term, high-dose and/or potent ICS use, or concomitant use of P450 inhibitors (such as ritonavir, ketoconazole, itraconazole) that can markedly increase both bioavailability and decrease clearance of most of the ICS (3). Ongoing monitoring of medication side-effects should be an essential component of a comprehensive childhood asthma management program.

## Uncontrolled asthma: difficult vs. severe asthma in children

The actual asthma management is control-based, including evaluation of symptom control and risk domains; therapeutic strategies are based on a stepwise approach and adjusted in a continuous cycle involving assessment, treatment and review (3). Asthma severity should be determined before the patient is treated, while the assessment of asthma control should be performed after treatment has been instituted; then, both of them should be re-determined at every visit. Step up and step down of treatment should be adapted to every patient in order to maintain asthma control with the minimum dose of medication. The frequency of the assessment of asthma control is variable and depends on disease activity but typically is every 1–6 months. Patients with asthma may be classified into three broad categories as well controlled, partly controlled, or uncontrolled, according to established criteria (Table 3) (3).

**Table 3:** Levels of asthma symptom control (to assess retrospectively in the past 4 weeks). adapted from Global Initiative for Asthma (3).

	**Well-controlled**	**Partly controlled**	**Uncontrolled**
Daytime asthma symptoms more than twice a week	None of these	1 or 2 of these	3 or 4 of these
Night waking due to asthma			
Need for reliever^*^ for symptoms more than twice a week			
Limitation of activity due to asthma			

* *Excluding before exercise*.

Identifying children and adolescents with uncontrolled severe asthma is important because they potentially need close monitoring and additional treatment with advanced biological therapies (78–82). Although accounting for <5% of all pediatric asthma (83), uncontrolled severe asthma carries the majority of morbidity and accounts for nearly 50% of all asthma healthcare costs, and even mortality (8). Children with persistent uncontrolled asthma, despite maximal therapy, are defined as having problematic severe asthma, an umbrella term comprising asthma-mimicking conditions, asthma that is difficult-to-treat because of comorbidities, improper inhaler technique or poor therapeutic adherence, and other environmental factors, and true severe therapy-resistant asthma, as defined by the latest European Respiratory Society/American Thoracic Society (ERS/ATS) definition (8, 84, 85): after confirming a diagnosis of asthma and addressing comorbidities, true severe therapy-resistant asthma is that which requires treatment with high dose inhaled glucocorticoids plus a second controller and/or systemic glucocorticoids to prevent asthma from becoming ‘uncontrolled’ or which remains 'uncontrolled' despite this therapy (8). The identification of the factors that may contribute to the gain or loss of control in asthma is essential in differentiating children with difficult-to-treat asthma from those with severe asthma that is resistant to traditional therapies.

### Monitoring and managing comorbidities

Once alternative diagnoses have been excluded and the diagnosis of asthma confirmed, the contribution of comorbid conditions to disease severity should be evaluated. Childhood asthma is associated with several comorbidities (Table 4), variably presenting and depending on the age of the subject (86–89). Observations from severe asthma international registries and cohort studies highlighted a distinct picture for pediatric age. In the National Heart, Lung and Blood Institute's Severe Asthma Research Program (SARP) the highest prevalence of comorbid conditions, namely sinus disease, gastroesophageal reflux and obesity, has been found in only 20% of children studied (clustered as “Group 3”) (90). In the Unbiased Biomarkers for the Prediction of Respiratory Disease Outcomes (U-BIOPRED), around 65% of school-aged children with severe asthma had a diagnosis of concomitant allergic rhinitis, while 40% of children reported food allergy (91). Unlike upper airway pathology, the relationship between gastroesophageal reflux and asthma is still a matter of debate and there is not enough evidence to support causality; besides, it has been demonstrated that treating reflux does not actually improve asthma outcomes (92). Likewise, the relationship between food allergy and respiratory symptoms is still unclear.

**Table 4:** Asthma comorbidities in childhood.

**Comorbid Conditions**	**Potential mechanism contributing to worsen asthma**
Obesity	Mechanical effects on lung functions; pro-inflammatory state contributing to airway inflammation; corticosteroid resistance
Gastroesophageal reflux	Direct contamination of the lower airway; esophago-bronchial reflex; reduced efficiency of the lower esophageal sphincter due to altered configuration of the diaphragm during respiratory disease
Food allergy	Unclear (consider anaphylaxis at rest and on exercise in the differential diagnosis)
Rhinosinusitis	Shared complex inflammatory mechanisms between upper and lower airways, according to the “United Airways Disease” concept
Upper Airway Obstruction/Sleep Disordered Breathing	Obesity-associated (common); increased neutrophilic inflammation of the airways
Dysfunctional Breathing	Unclear (also consider vocal cord dysfunction and other hyperventilation syndromes in the differential diagnosis)

Dysfunctional breathing has been reported as common in some asthma cohort; however, the available data suggest that 5.3% or more of children with asthma have dysfunctional breathing and that, unlike in adults, it is associated with poorer asthma control (93). Besides, the results of an observational cohort study conducted on 71 children with problematic asthma reported that 15% of them has dysfunctional breathing, including hyperventilation and vocal cord dysfunction (94). Although it is not possible to draw conclusive estimates on the prevalence of comorbidities in childhood severe asthma, it is well established that the presence of one and/or more comorbid conditions contributes to the loss of asthma control and may complicate the assessment of asthma and potentially impact on its management, outcome and healthcare expenditure (8, 86, 88). Thus, addressing comorbidities is an essential step in the approach to the child with uncontrolled asthma.

### Adherence to treatment

Determining if the child is receiving the prescribed controller medication with the adequate inhaler technique is another essential cornerstone of the management of childhood asthma. Poor adherence to treatment is common in asthmatic children, as well as in adults, with reported rates varying from 30 to 70% (85, 94, 95) and it should be considered in all children with uncontrolled asthma. Medication issues do not only concern about therapeutic adherence, but also affect management of prescriptions, parental supervision, and use of inhaler devices (70, 94, 96). These are all potentially modifiable factors that have been associated with loss of asthma control (97). Interventions to improve patient adherence have been tested in several clinical trials and demonstrated a variable efficacy (94). Identifying non-adherent patients and establishing an educational intervention can be complex and time-consuming and require an integrated strategy in everyday clinical practice including regular follow-up assessment at the doctor office, effective patient-centered behavioral interventions, improvement of doctor-patient communication, and new instruments of control such as electronic monitoring (85).

### Review of environmental factors

Ongoing exposure to environmental factors is an important cause of poor asthma control in some patients (98). For patients with severe asthma who have an allergic component to their disease, allergen control measures will have an important effect. Potential triggers should be identified during evaluation and need renewed strategies for control. Common inhalant allergens that can contribute to poor asthma control and cause attacks include animal allergens (both pets and pests: cats, dogs, rodents), house dust mites, cockroaches, indoor and outdoor fungi and outdoor plant allergens (tree, grass, ragweed pollen) (99–101). In particular, home and school exposures to pets, house dust mites, mold are associated with severe asthma attacks in children, supporting the importance of atopy in this population (98).

Fungal exposure and sensitization have been recently associated with a sub-phenotype of childhood asthma characterized by increased disease severity, AHR, airway eosinophilic inflammation, more exacerbations and relative steroid resistance (102). Severe asthma with fungal sensitization (SAFS) is a recognized subphenotype of severe asthma in both children and adults. SAFS is defined as severe, therapy-resistant asthma with fungal sensitization demonstrated by a positive skin prick test response or specific IgE to at least one of 7 fungi (i.e., Aspergillus fumigatus, Alternaria alternata, Cladosporium herbarum, Penicillium chrysogenum, Candida albicans, Trichophyton mentagrophytes, or Botrytis cinerea), with IgE <1,000 and with negative IgG Aspergillus serology (102). Little is known about the mechanisms mediating this subphenotype. Recent evidence suggests a role for innate epithelial cytokine IL-33 in the pathogenesis of SAFS, as higher levels of IL-33 have been found on BAL and airway samples from children with SAFS compared to children without SAFS (102). Till date there are no guidelines for the diagnosis and treatment of SAFS in pediatric population: reduction of environmental fungal exposure in the home and antifungal therapy have been anecdotally successful in some children.

There are also non-allergic factors that potentially contribute to asthma such as exposure to tobacco smoke (including active and passive smoking, as well as vaping), environmental pollution and other irritants (i.e., incense, joss sticks, air fresheners and other aerosol sprays) (103). Tobacco smoke exposure is common in children with asthma and it has been reported in 25% of cases in a cohort of children having problematic severe asthma (94); it is also associated with loss of asthma control, higher incidence of respiratory infections, increased likehood of asthma attack-related hospitalizations (104–107); furthermore, it was found that parental (passive) smoking impairs histone deacetylase-2 function, which could contribute to increased corticosteroid-resistant inflammation in children with severe asthma (108). In addition, exposure to tobacco smoke exacerbates inflammatory airway responses to allergens (109). Exposures at home, daycare, school and/or work should be reviewed.

### Evaluation of psychosocial factors

Asthma attacks may be triggered by both acute and chronic stress, through a supposed effect of enhancement of allergic eosinophilic airway inflammatory response (110). Among psychosocial issues, anxiety and depression have been more frequently reported in children with persistent and severe asthma rather than mild or intermittent asthma, and in their families (94, 111, 112). Given the complexity of relationship between these two entities, it is yet to be determined whether anxiety and depression are the cause or result of severe asthma; however, both should be treated on their individual merits.

## Conclusions

Over the past 10 years, there has been an increasing interest on the concept of asthma control, with development and validation of new and promising “asthma control tools.”

Uncontrolled persistent asthma in children represents a clinical challenge and requires a multidisciplinary systematic assessment, including the assessment of comorbid conditions, treatment- related issues, environmental exposures and psychosocial factors. The presence or absence of these factors may contribute to the gain or loss of control and differentiate children with difficult-to-treat asthma from those with true severe therapy-resistant asthma. Finally, the early identification of modifiable factors contributing to childhood uncontrolled asthma is essential to avoid a further and useless escalation of treatment; likewise, addressing a correct diagnosis of true severe therapy-resistant asthma avoids deferring of invasive testing and advanced biological therapies.

## Author contributions

All authors made substantial contribution to the conception of the work, reviewed the literature on the subject, and drafted the final version of the manuscript; AL, VP, and GM revised it critically for important intellectual content. All authors finally approved the version to be published and agreed to be accountable for all aspects of the work in ensuring that questions related to the accuracy or integrity of any part of the work are appropriately investigated and resolved.

### Conflict of interest statement

The authors declare that the research was conducted in the absence of any commercial or financial relationships that could be construed as a potential conflict of interest.

## Footnotes

^1^Data from National Review of Asthma Deaths' (2014) Available online at: https://www.rcplondon.ac.uk/projects/outputs/why-asthma-still-kills.
